# The impact of a single HIIT intervention on the mobilization of NK cells and ILCs in adolescents and young adults (AYA) undergoing cancer treatment: an interventional controlled trial

**DOI:** 10.1186/s12885-025-14058-3

**Published:** 2025-04-14

**Authors:** Isabella Deppe, Ronja Beller, Fabian Kiehl, Nico De Lazzari, Sabrina B. Bennstein, Dirk Reinhardt, Uta Dirksen, Miriam Götte

**Affiliations:** 1 Pediatrics III, West German Cancer Center, University Hospital Essen, University Duisburg- Essen, 45147 Essen, Germany; 2 West German Cancer Center, University Hospital Essen, 45147 Essen, Germany; 3 Institute for Transplantation Diagnostics and Cell Therapeutics, Medical Faculty and University Hospital Düsseldorf, Heinrich-Heine University Düsseldorf, 40225 Düsseldorf, Germany; 4 German Cancer Consortium (DKTK), site Essen, National Center for Tumourdiseases (NCT) site Essen, University Hospital Essen, 45147 Essen, Germany; 5 Department of Palliative Medicine, West German Cancer Center Essen, University Hospital Essen, 45147 Essen, Germany; 6 Institute of Immunology, Faculty of Medicine, RWTH Aachen University, 52074 Aachen, Germany; 7 GPOH gGmbH, Essen, Germany

**Keywords:** Natural killer cells, Innate lymphoid cells, Sarcoma, Oncology, Physical activity, AYA

## Abstract

**Objective:**

The study investigated the response of immune cells, particularly natural killer (NK) cells and innate lymphoid cells (ILCs), to acute exercise in adolescents and young adults (AYAs) undergoing cancer treatment, to lower their treatment burden and evaluate the value of exercise in this vulnerable cohort.

**Methods:**

An AYA cancer patient group (PG) (*n* = 20, 25 ± 7 years old) and an age-matched healthy control group (HG) (*n* = 20, 27 ± 5 years old) completed a twenty-minute high intensity interval training (HIIT) on a bicycle ergometer. Blood was taken at three timepoints during the intervention. Once immediately before (T0), once immediately after the intervention (T1), and after one-hour of recovery (T2). NK cells, ILCs, respectively their subpopulations, were determined by flow cytometry.

**Results:**

Total NK cells (PG: *p* = 0.023; HG: *p* = 0.004), CD56^dim^NK cells (PG: *p* = 0.035; HG: *p* = 0.004), total ILCs (PG: *p* < 0.001; HG: *p* < 0.001), ILC1-like (PG: *p* = 0.001; HG: *p* = 0.004), ILC2 (PG: *p* = 0.006; HG: *p* = 0.003) and innate lymphoid cell precursors (ILCPs) (PG: *p* = 0.009; HG: *p* = 0.002) increased significantly from T0 to T1. CD56^bright^NK cells (HG: *p* = 0.011) increased significantly only in the HG. From T1 to T2 total NK cells (PG: *p* < 0.001; HG: *p* < 0.001), CD56^dim^NK cells (PG: *p* < 0.001; HG: *p* < 0.001), CD56^bright^NK cells (PG: *p* < 0.001; HG: *p* < 0.001), ILC2 (PG: *p* = 0.035; HG: *p* = 0.007) and ILCPs (PG: *p* = 0.006; HG: *p* = 0.003) decreased significantly. ILC1-like maintained their elevated cell count plateau during the recovery phase. No significant differences were found for NKp44^+^ILC3 and for inter-group comparisons regarding the percentage changes of cell counts from T0 to T1 or T1 to T2. Younger age and higher heart rates (in percentage of age-predicted maximal heart rate) during the intervention were associated with an increased mobilization of immune cells, especially in NK cells and their subpopulations.

**Conclusion:**

We were able to show, that HIIT enhances the mobilization of NK cells and ILCs to the same extend in AYA cancer patients than in healthy controls. Our pilot study revealed, that exercise is likely to play an important role in the defense against pathogens and neoplastic cells and that AYA cancer patients might benefits from regular exercise programs during anti-cancer treatment.

**Trial registration:**

: The study was registered on 13.11.2022, registration number NCT05656651, in the international register of clinical trials https://www.clinicaltrials.gov/.

**Supplementary Information:**

The online version contains supplementary material available at 10.1186/s12885-025-14058-3.

## Introduction

Worldwide 1,355,100 new cancer diagnoses were registered 2019 in AYA (adolescent and young adults) cancer patients [1] (which are defined as cancer diagnosed between the ages of ≥ 15 and ≤ 39 [2]). Among these, common cancer types are not carcinomas as in adults, but lymphoma, leukemia and sarcoma [2, 3] with increasing incidence [4]. Recent studies suggest, that AYA patients are at higher risk of long-term and late effects compared to pediatric cancer patients due to different tumor biology [5, 6]. AYAs diagnosed with rhabdomyosarcoma are 22.8% less likely to achieve an event free survival of five years, or 26% less likely to attain a five-year overall survival, compared to children with the same diagnosis [7]. For acute myeloid leukemia, it has been shown that AYA patients have a higher treatment-related mortality than younger patients [8]. Next to cancer-specific mortalities AYA cancer patients suffer from higher non-cancer mortality e.g. infection-related mortality, due to late effects of the anticancer treatment [9]. Research on supportive therapy is crucial due to physical and emotional burdens imposed on these young patients. Cancer and anti-tumor treatment weaken the immune system and make patients more vulnerable to pathogens. Therefore, fast-acting immune cells, including natural killer (NK) cells, but also innate lymphoid cells (ILCs), are of outstanding importance [10]. Positive impacts of physical activity have been reported in healthy cohorts and cancer patients [11–13]. Besides enhancing physical fitness, fatigue and quality of life [14–17], exercise potentially reduces cancer development, mortality, and metastasis growth [18–21]. The mechanisms by which exercise influences tumor growth are currently the focus of many investigations and remain an active area of research. Exercise exerts anti-tumor effects through multiple pathways, including improving tumor perfusion and reducing hypoxia, which enhances treatment efficacy [22]. It also lowers systemic inflammation and reduces pro-tumor cytokines like IL-6 and TNF-α [23]. Additionally, exercise-derived myokines have shown to inhibit cancer cell proliferation [24]. The innate immune system, and in particular NK cells, appear to play an important role in this context [18].

CD56^+^NK cells, including CD56^bright^NK cells and CD56^dim^NK cells, are innate immune cells [10] which exhibit natural cytotoxicity against neoplastic cells and pathogens by recognizing the absence of human leukocyte antigen (HLA) class I molecules [25]. CD56^bright^NK cells predominantly secrete cytokines, and have a regulatory function. The high cytotoxicity of CD56^dim^NK cells [26] is triggered, among others, by the recognition and elimination of target cells via the antibody-dependent cell-mediated cytotoxicity (ADCC) pathway. In further process they release IFN-y or other cytotoxic substances, such as perforin and granzymes, which results in lysis and thus in apoptosis of target cells [27]. It is known that CD16 expression is essential for the ADCC and CD16 expression is down-regulated upon activation of NK cells [28]. Indirectly, therefore, it might be speculated that reduced CD16 expression on NK cells during a single bout of exercise indicates the activation of NK cells. NK cell mobilization into the circulation through exercising has extensively been studied in healthy people [29, 30] and is recently also demonstrated for cancer patients [12, 13, 31]. The extend of immune cell mobilization appears to correlate with the exercise intensity, with most significant effects observed in the high intensity range [32]. Innate lymphoid cells (ILCs), including ILC1, ILC2, and ILC3, contribute to immune defense and tissue homeostasis, primarily at barrier sites [33, 34]. Circulating ILC1-like cells and innate lymphoid precursors (ILCPs) are able to differentiate into NK cells or other ILC subsets [35, 36]. They may migrate into tissues to replenish immune cell populations. While ILC1-like cells enhance immune surveillance by inducing apoptosis, e.g. in neoplastic cells [37], the response of ILC2 depends on cytokine secretion, which can have variable effects on tumors [38]. NKp44 + ILC3, rarely found in healthy individuals [35], have been detected in cancer patients undergoing chemotherapy or stem cell transplantation [39], where they may support tissue repair. Given their role in immunity and tissue regeneration, evaluating ILCs in the context of cancer and exercise is of particular interest. The role of circulating ILCs in cancer patients, and their response to exercise especially in cancer patients is still not well known, but research in healthy people have shown, that they can be mobilized through exercising [40]. Nevertheless, due to their ability to secrete cytokines, it can be hypothesized that ILCs play an essential role in changes within tumor microenvironment and in controlling patient’s susceptibility to infections.

Previous studies observing increased mobilization of NK cells mainly included adults or younger children [13, 41–43]. Research focusing on AYAs is essential, as the effects observed in adults and children cannot be directly applied to AYAs due to the differences in tumor biology and immune function. Furthermore, there is a lack of research that compares the mobilization of immune cells in cancer patients to healthy controls to evaluate potential differences in immunological response to exercise during cancer treatment. Therefore, the aim of this study was to investigate the acute response of immune cells, especially NK cells, ILCs and their respective subgroups, to a single session of high-intensity-interval-training (HIIT) in AYA cancer patients and healthy controls, to enable a direct comparison between these two groups.

## Methods

### Study design

This monocentric interventional trial included an AYA cancer patient group (PG) and an individually matched healthy control group (HG). Patients were screened at a tertiary care university hospital for eligibility and informed about the study between November 2022 and May 2023. The HG was recruited and matched based on the following criteria: (1) gender, (2) age ± 5 years, (3) body mass index (BMI) ± 5 kg/m^2^, (4) interests in sport ± 3 points (scale from 1 to 10). The study is registered in the international register of clinical trials (NCT05656651– registration date 13.11.2022) and approved by the local Ethics Committee (19-8789-BO). Blood was collected at three time points: directly before the intervention (T0), directly after (T1), and after one-hour of recovery (T2). Primary aim was to analyze changes in circulating cell counts of NK cells, ILCs and their subsets, between T0 and T1 in PG versus HG. Secondary endpoints were changes in peripheral cell counts during a one-hour resting period after the acute exercise, correlations of exercise-induced cell mobilization with clinical or exercise parameters and minor functionality analysis regarding altered CD16 expression on CD56^dim^NK cells between the time points.

### Participants

We aimed to recruit 20–25 AYAs, and a corresponding number of age-matched control individuals. Inclusion criteria for the PG were (1) age ≥ 15 and ≤ 39 years, (2) current curative cancer therapy, (3) ≥ 1 completed cycle of chemotherapy, (4) signed informed consent. We excluded patients with (1) medical contraindication for HIIT on the bicycle ergometer (model: Bike 1000 MED, Technogym, Italy; sitting bicycle ergometer: RECLINE 1000 MED, Technogym, Italy), (2) immunotherapy or (3) being unable to follow instructions due to cognitive disabilities or language barriers. Medical contraindication for the single bout of exercise intervention were evaluated by the responsible physician and included, for example, an increased risk for pathological fractures due to the tumor itself or metastases, increased cardiotoxicity of some chemotherapeutic agents, or limitations in the range of motion or loading restrictions of extremities or spine due to surgery. HG could be included if they (1) fulfilled the matching criteria, (2) did not suffer from preexisting diseases and (3) signed informed consent.

### Acute exercise intervention

Pretesting requirements for the start of the acute exercise intervention were (1) patient had not received chemotherapy within the past 48 h, (2) hemoglobin level was ≥ 8 mg/dl and (3) thrombocyte count was ≥ 20.000/µl [44]. We aimed to conduct an intervention in the high-intensity range, as prior research indicates that within this intensity spectrum, the acute effects of exercising on immune cells in healthy individuals are most significant. Olofsson et al. [45] successfully performed HIIT in lung cancer patients during acute oncological treatment, allowing us to assume that the intensity of the exercise is manageable for the patients. However, a standardized 20-minute high-intensity endurance intervention comparable with healthy controls, could be difficult to achieve for cancer patients and could lead to exhaustion and a high rate of drop-outs. Consequently, we have opted for interval training, specifically HIIT. This resulted in interventions that are comparable to those of healthy controls in terms of intensity, duration and exertion. We defined a perceived exertion of 15–17 on the RPE scale (RPE scale 0–20) as the superior target [46, 47]. Additionally, we set different heart rate ranges (HR) as secondary targets. Intervention and target values are provided in the supplemental file (S1). It started with a 7-minute warm-up period at 30 watts, followed by a HIIT period comprising 6 cycles, each of 30 s high intensity and 60 s recovery, and a wattage that was aimed to correspond to 1-1.5x the body weight (kg). A 4-minute cool-down was completed at 30 watts. HR was monitored using Polar H10 Heart Rate Sensor and oxygen saturation was measured to ensure saturation of ≥ 90%. Additionally, rotations per minute were recorded by the ergometer during warm-up and cool down every minute and during the HIIT once during each intensity and recovery phase.

### Questionnaires

Questionnaire data were collected during recovery. With the Mini Nutritional Assessment Questionnaire, the nutritional status of the participants was determined. A demographic questionnaire collected data about age, gender, employment status, current interest in sport and before the diagnosis. The EORTC QLQ-C30 assessed participants’ health-related quality of life (QoL) and general Fatigue status.

### Blood sampling

Blood was collected in sterile 2.5 ml EDTA-tubes at T0, T1 and T2 via central venous catheter or from antecubital vein. EDTA blood was stored at room temperature until preparation for flow cytometry and actual flow cytometry was performed within maximum 24 h. Participants were instructed not to eat during the recovery but were allowed to drink water.

### PBMC isolation and staining

Differential blood count and hematocrit was determined by using Sysmex (model XN-1000) for the respective time points. Subsequently, EDTA blood was diluted 1:1 with sterile Phosphate-Buffer-Saline (Dulbecco’s Phosphate Buffered Saline, DPBS). Peripheral blood mononuclear cells (PBMCs) were isolated by density gradient centrifugation (Ficoll plaque PLUS) without breaks. Any remaining red blood cells within the PBMCs were lysed (BD Pharm Lyse, BD Biosciences) and cells were washed twice with DPBS. For epitope blocking, cells were washed with DPBS containing 0.5% BSA (Roth) and 5 mM EDTA (Gibco™) and immediately used for analysis. An antibody master mix (supplemental file S2) was added to stain NK cells and ILCs and incubated for 20 min in a dark refrigerator. Stained cells were washed, centrifuged, and stored cold and dark until flow cytometry analysis was performed on a Cytoflex (Beckman Coulter).

### Analysis of flow cytometry & gating strategy

**Fig. 1:**
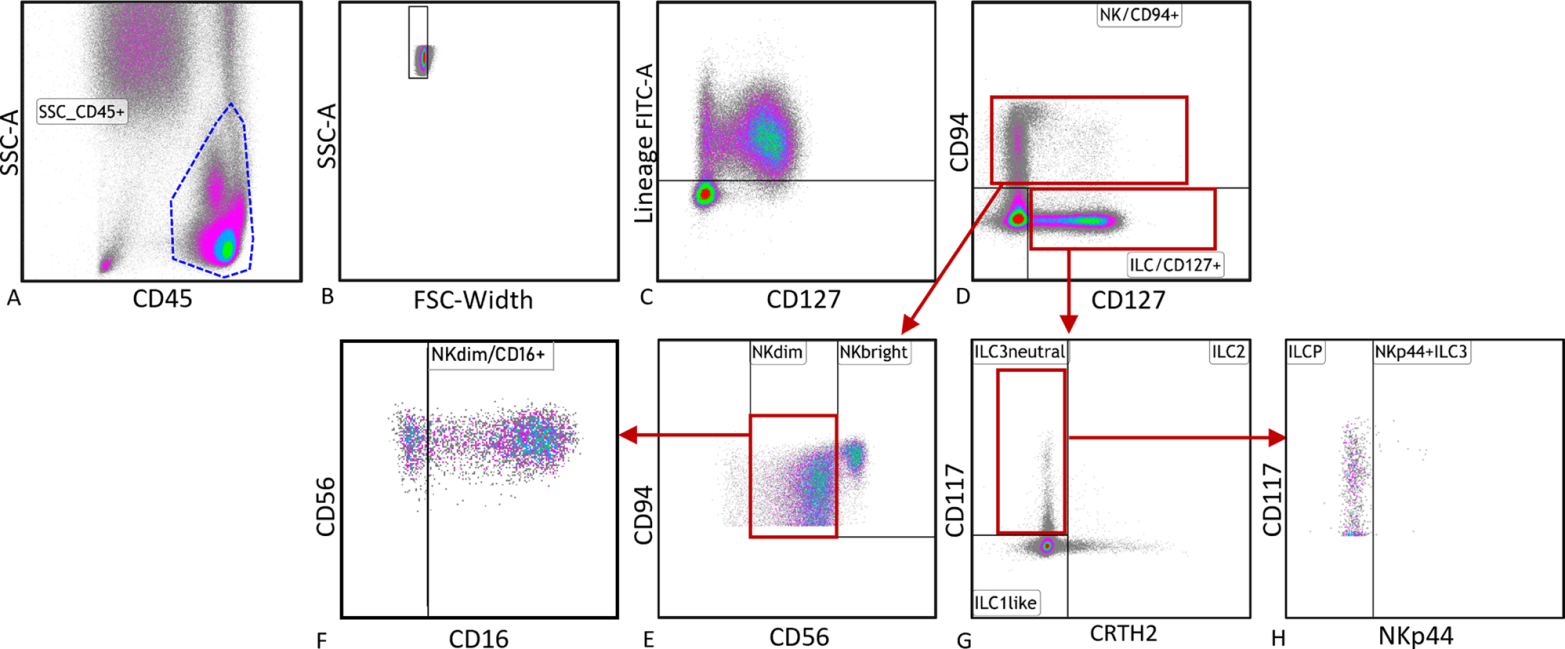
Flow cytometry gating strategy. (**A**) Selection of CD45^+^ lymphocytes, (**B**) gating of single cells, (**C**) excluding lineage positive cells, (**D**) differentiation of NK cells and ILCs, (**E**) differentiation of NK cells (CD94^+^, CD56^+^) and their subpopulations CD56^bright^NK cells (CD94^+^, CD56^++^) and CD56^dim^NK cells (CD94^+^, CD56^+^), (**F**) CD16 positive CD56^dim^NK cells. (**G**) differentiation of ILCs (CD94^−^, CD127^+^) and their subpopulations ILC1-like cells (CD117^−^, CRTH2^−^), ILC2 (CD117^+/−^, CRTH2^+^) and ILC3 neutral (CD117^+^, CRTH2^−^), (**H**) subdivision of ILC3neutral into ILCPs (NKp44^−^) and NKp44^+^ ILC3

The gating commenced with the selection of single CD45^+^lymphocytes (Fig. 1A + B). Lineage-positive cells were excluded from further analyses (Fig. 1C). NK cells were identified using CD94 antibody, while ILCs were identified using CD127 (Fig. 1D). NK cells were further categorized into CD56^bright^ and CD56^dim^NK cells based on their CD56 expression (Fig. 1E). Indirect assessment of NK cell functionality was done via expression of CD16 on CD56^dim^NK cells (Fig. 1F). ILCs were subdivided into ILC1-like (CD117^−^, CRTH2^−^), and ILC2 (CD117^+/−^, CRTH2^+^), and ILC3 neutral (CD117^+^, CRTH2^−^) based on their expression of CD117 and CRTH2 (Fig. 1G). NKp44 was used to subdivide ILC3 neutral cells into NKp44^+^ILC3 and ILCP, which are NKp44^−^ (Fig. 1H). Based on the methodology and reference values published by Uyen Pham et al. 2022 [48], the final cell counts from flow cytometry were calculated as follows:

$$\:\frac{\:\#\:cell\:count\:\left(cytoflex\right)}{\#\:lymphocytes\:\left(cytoflex\right)}\text{*}lymphocyte\:cell\:count\:\left[x{10}^{3}\:pro\:{\upmu\:}l\right]\:\left(Sysmex\right).$$

### Statistical analysis

Due to the exploratory nature of this study, we did not perform a formal sample size calculation. Instead, we set a minimum of 40 participants (20 AYAs and 20 healthy controls) based on a realistic recruitment estimate and the commonly recommended threshold for exploratory statistical analyses [49]. Flow cytometry data were analyzed with Kaluza (Beckmann Coulter, version 2.2). Normal distribution was assessed with a Shapiro-Wilk test. Since no normal distribution was found here, we used the non-parametric Friedman test to evaluate the absolute changes in cell counts between T0 vs. T1, and T1 vs. T2 within the groups. Dunn-Bonferroni posthoc pairwise comparisons identified statistical differences between the time points. With the Mann-Whitney-U-test differences were analyzed between the PG vs. HG regarding percentual changes of mobilized immune cells from T0 to T1, and T1 to T2. A Spearman’s rank correlation was used to detect correlations between immune cell mobilization and BMI, age, gender, and maximal reached HR (%) of age-predicted HR. In the correlation analyses, the correlation coefficient is categorized as: 0.10–0.29 = weak correlation, 0.30–0.49 = moderate correlation and > 0.50 = strong correlation. Statistical significance was set at *p* < 0.05 for all tests, and statistical analyses were conducted using IBM SPSS version 28.0.1.1. All graphs were generated with GraphPad Prism 10.

## Results

### Participants characteristics

**Fig. 2:**
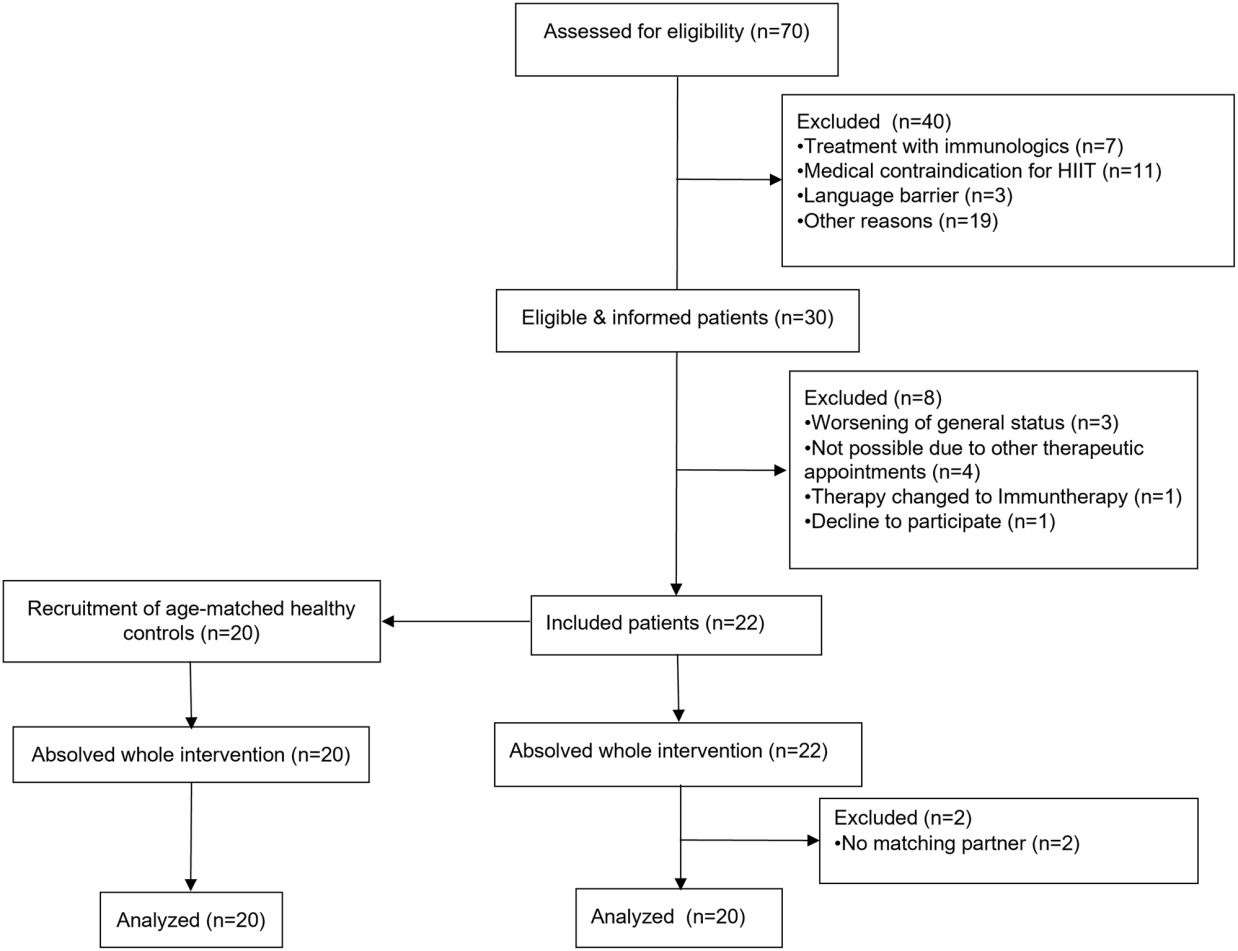
Screening and recruitment of the participants with reasons for exclusion

Out of *n* = 70 patients screened, *n* = 30 (43%) fulfilled the inclusion criteria and thereof *n* = 22 (73%) performed this one-time intervention (Fig. 2). Twenty patients (25 ± 7 years old) were analyzed, including 12 males, 8 females with a mean BMI of 22.7 ± 5.2 kg/m². Their interests in sports before diagnosis was 7 ± 2 (scale from 1 to 10, 0 = no interests, 10 = great interest). Further details on diagnoses and treatment are given in Table 1. In accordance with these patients, 20 healthy controls were included. Matching criteria resulted in the following characteristics: 12 males and 8 females, 27 ± 5 years old, BMI 23.4 kg/m^2^ ± 4.4 and interests in sports 8 ± 1.

**Table 1:** Participant characteristics and data of intervention

			Patients (*n* = 20)	Healthy controls (*n* = 20)
Age (years)			25 **±** 7	27 **±** 5
Gender N(%)	Male		12 (60)	12 (60)
	Female		8 (40)	8 (40)
Body-Mass-Index kg/m²			22.7 **±** 5.2	23.4 **±** 4.4
Nutritional status (MNA Questionnaire) N(%)	Normal nutritional status		9 (45)	16 (80)
	Risk for malnutrition		8 (40)	4 (20)
	Malnutrition		3 (15)	-
Interests in sport before diagnosis*			7 **±** 2	-
Interests in sport at present*			6 **±** 2	8 **±** 1
Diagnosis N(%)	Sarcoma		16 (80)	-
		Ewing-Sarcoma	8 (50)	
		Round-cell Sarcoma	4 (25)	
		Synovial-Sarcoma	2 (12.5)	
		Osteoblastic Osteosarcoma	1 (6.25)	
	Lymphoma		3 (15)	
		Hodgkin-Lymphoma	1 (33)	
		T-lymphoblastic Lymphoma	2 (66)	
	Epitheloid Hemangioethelioma		1 (5)	
Localization of sarcoma N(%)	Upper extremity		3 (18.5)	-
	Thorax		1 (6.25)	
	Pelvis		4 (25)	
	Lower extremity		1 (6.25)	
	Soft tissue		3 (18.75)	
	Visceral		2 (12.5)	
	Spine		2 (12.5)	
Relapse N(%)	No		16 (80)	-
	Yes		4 (20)	
Metastases N(%)	No		10 (50)	-
	Yes		10 (50)	
UICC stadium N(%)	II		4 (33.3)	
	III		3 (25)	-
	IV		5 (41.7)	
Previous cycles of chemotherapy			4 **±** 3	-
Health related QoL			56.6 **±** 21.5	85.8 **±** 9.9
Fatigue			43.8 **±** 25.9 (*n* = 19)	11.1 **±** 10.5
Ø HR (bpm)	HIIT		151 ± 20	146 ± 18
		Intensity	152 ± 21	148 ± 19
		Recovery	150 ± 20	142 ± 17
	Total		141 ± 18	125 ± 15
Maximal reached HR % from age-predicted maximal HR			84.26 ± 9.26	85.36 ± 9.78
RPE HIIT			17 ± 1	16 ± 1
Reached HR and RPE target values** N(%)	No		11 (55)	8 (40)
	Yes		9 (45)	12 (60)
Ø Watt power HIIT			56.55 ± 25.64	118.48 ± 36.08
Ø RPM	HIIT		70.72 ± 17.14	102.78 ± 17.12
	Intensity		87.94 ± 24.16	130.41 ± 26.28
	Recovery		52.32 ± 12.45	74.60 ± 16.42
	Total		64.24 ± 15.60	90.21 ± 14.68
Adverse events N(%)	No		20 (100)	20 (100)
	Yes		0 (0)	0 (0)

* On a scale from 0 to 10 (0 = no interests, 10 = great interest)

** Target values: RPE 15–17, 85% of maximal HR (in percentage from age predicted HR)

Data are reported as mean (SD) or count (proportion). Assessment of health related QoL and Fatigue was done with EORTC QLQ-C30 (range from 0-100, higher scores represent high QoL or high level of Fatigue). Adverse events during the intervention: e.g. vomiting, low oxygen supply in the blood or exhaustion of the patients before protocol was finished

Abbreviations: SD = standard deviation; n = number of participants; UICC = union internationale contre le cancer; HR = heart rate; RPE = rate of perceived exertion on RPE Scale; RPM = revolutions per minute; HIIT = high intensity interval training; bpm = beats per minute

### Exercise characteristics

Table 1 shows that the HR reached during different parts of the intervention, and the maximum achieved HR (%) of age-predicted maximum HR during the HIIT, were similar between the groups (PG: 84.3 ± 9.3%; HG: 85.4 ± 9.8%). To reach an equal RPE range, watt power in the HG was twice as high as in the PG (PG: 56.6 ± 25.6; HG: 118.5 ± 36.1). The PG reported for the same intensity an average of 17 ± 1 for their exertion during HIIT on the RPE scale, versus 16 ± 1 for the HG. In the PG, nine participants achieved the prescribed thresholds for sufficient exertion (defined as 15–17 on RPE scale and a minimum of 85% of age-predicted maximal HR). In the HG twelve participants attained both target values.

### Mobilization of NK cells and ILCs

#### Change in hematocrit

The median hematocrit change was 0.01% from T0 to T1 and − 0.02% from T1 to T2 (supplemental file S3).

**Fig. 3:**
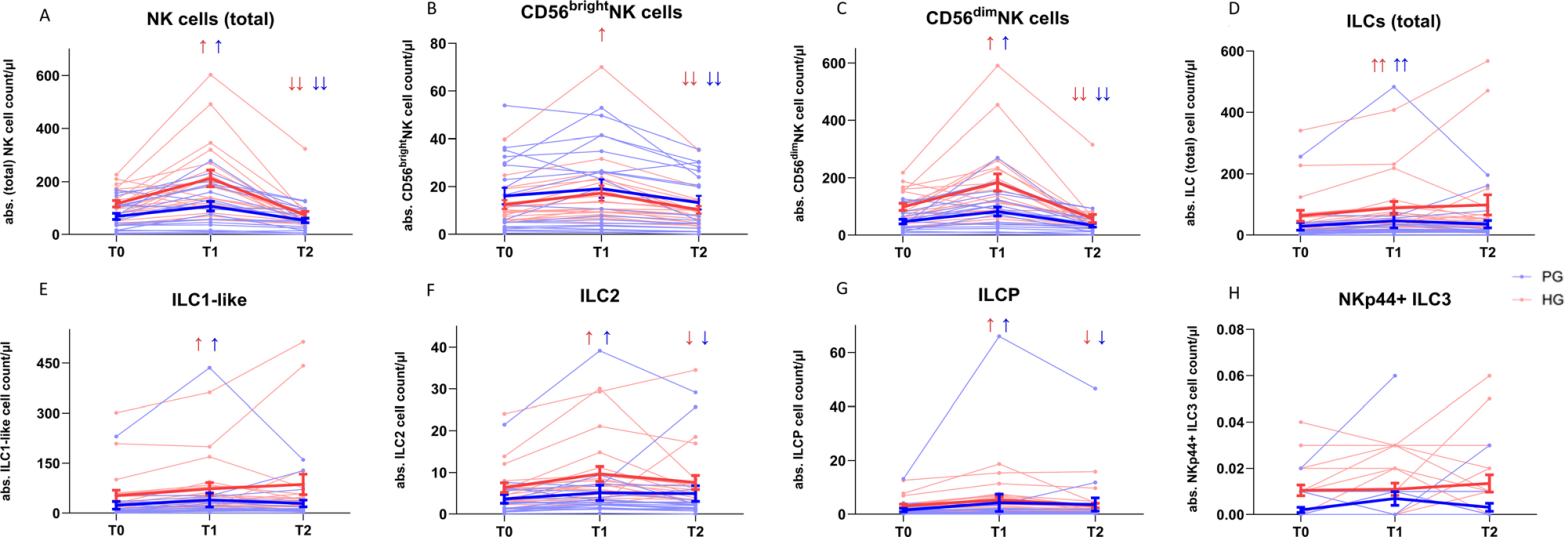
Absolute cell counts/µl at the three measurement time points. NK cells (**A**), their subpopulations CD56^bright^NK cells (**B**), CD56^dim^NK cells (**C**), total innate lymphoid cell count (**D**), their subpopulations ILC1-like (E), ILC2 (F), ILCP (G) and NKp44 + ILC3 (H) for the PG (blue, *n* = 20), HG (red, *n* = 20), represented as individual data (thin lines) and mean value (bold lines) with standard deviation. T0 = pre-exercise, T1 = post-exercise, T2 = after one hour of recovery. ↑/↓=*p* < 0.05; ↑↑/↓↓=*p* < 0.001

*Mobilization of NK cells within groups*: Total NK cell counts (Fig. 3A) revealed significant changes during the intervention (PG: *p* < 0.001, *n* = 19; HG: *p* < 0.001, *n* = 20). Following exercise, NK cell counts significantly increased (T0/T1 PG: 18.47, z=-0.737, *p* = 0.023; HG: 55.67, z=-0.9, *p* = 0.004) and subsequently decreased during recovery (T1/T2 PG: -17.72, z = 1.474, *p* < 0.001; HG: -105.55, z = 1.8, *p* < 0.001). A significant difference was also observed for CD56^bright^NK cells (Fig. 3B) (PG: *p* < 0.01, *n* = 19, HG: *p* < 0.001, *n* = 20). For the PG only the decrease during the recovery was significant (T1/T2 -1.99, z = 1.16, *p* < 0.001). In the HG the increase during exercise (T0/T1 2.48, z=-0.8, *p* < 0.001) changed significantly, as well as the decrease during recovery (T1/T2 -3.57, z = 1.75, *p* < 0.001). For CD56^dim^NK cells (Fig. 3C), significant differences were also demonstrated in both groups (PG: *p* < 0.001, *n* = 19; HG: *p* < 0.001, *n* = 20). This cell population revealed a significant increase in cell counts after exercise (T0/T1 PG: 16.40, z=-0.68, *p* = 0.035; HG: 51.40, z=-0.90, *p* = 0.004). The number of CD56^dim^NK cells also significantly decreased after recovering (T1/T2 PG: -15.12, z = 1.53, *p* < 0.001; HG: -91.90, z = 1.8, *p* < 0.001).

The analysis of the expression of CD16 on CD56^dim^NK cells did not reveal any significant differences between any of the measurement time points for the PG (*n* = 20). There were also no significant results for the HG (*n* = 20) from T0 to T1. From T1 to T2, however, a significant decrease in CD16 expression could be observed (*p* = 0.003).

*Mobilization of ILCs within groups*: Like NK cells, total ILCs (Fig. 3D) changed significantly in both groups (PG: *p* < 0.001, *n* = 19; HG: *p* = 0.004, *n* = 20). They significantly increased during exercise (T0/T1 PG: 6.20, z =-1.26, *p* < 0.001; HG: 18.07, z=-1.05, *p* < 0.001). However, ILC cell counts from T1 to T2 did not reveal any significant differences. ILC1-like cells (Fig. 3E) (PG: *p* < 0.001, *n* = 19; HG: *p* = 0.015, *n* = 20) showed significant increased mobilization during the HIIT (T0/T1 PG: 4.13, z=-1.16, *p* = 0.001; HG: 15.68, z=-0.9, *p* = 0.004). The same was observed for ILC2 (Fig. 3F) (PG: *p* = 0.016; *n* = 19; HG: *p* = 0.004, *n* = 20), with a significant increase during the intervention (T0/T1 PG: 0.41, z=-0.89, *p* = 0.006; HG: 1.78, z=-0.95, *p* = 0.003). Like NK cells, ILC2 significantly decreased during recovery (T1/T2 PG: -0.46, z = 0.68, *p* = 0.035; HG: -2.24, z = 0.85, *p* = 0.007). ILCPs (Fig. 3G) also changed significantly in both groups (PG: *p* = 0.008, *n* = 19; HG: *p* = 0.002, *n* = 20). They significantly increased during exercise (T0/T1 PG: 0.13, z=-0.84, *p* = 0.009; HG: 1.25, z=-1, *p* = 0.002) and decreased afterwards (T1/T2 PG: -0.10, z = 0.89, *p* = 0.006; HG: -1.30, z = 0.95, *p* = 0.003). NKp44^+^ILC3 (Fig. 3H) were detectable in some individuals but showed no significant changes between the time points in any group. The absolute cell counts of the respective cell population at the various measurement time points and their proportion in lymphocytes are shown in the supplementary material (S4).

#### Immune cell response between the groups

**Fig. 4:**
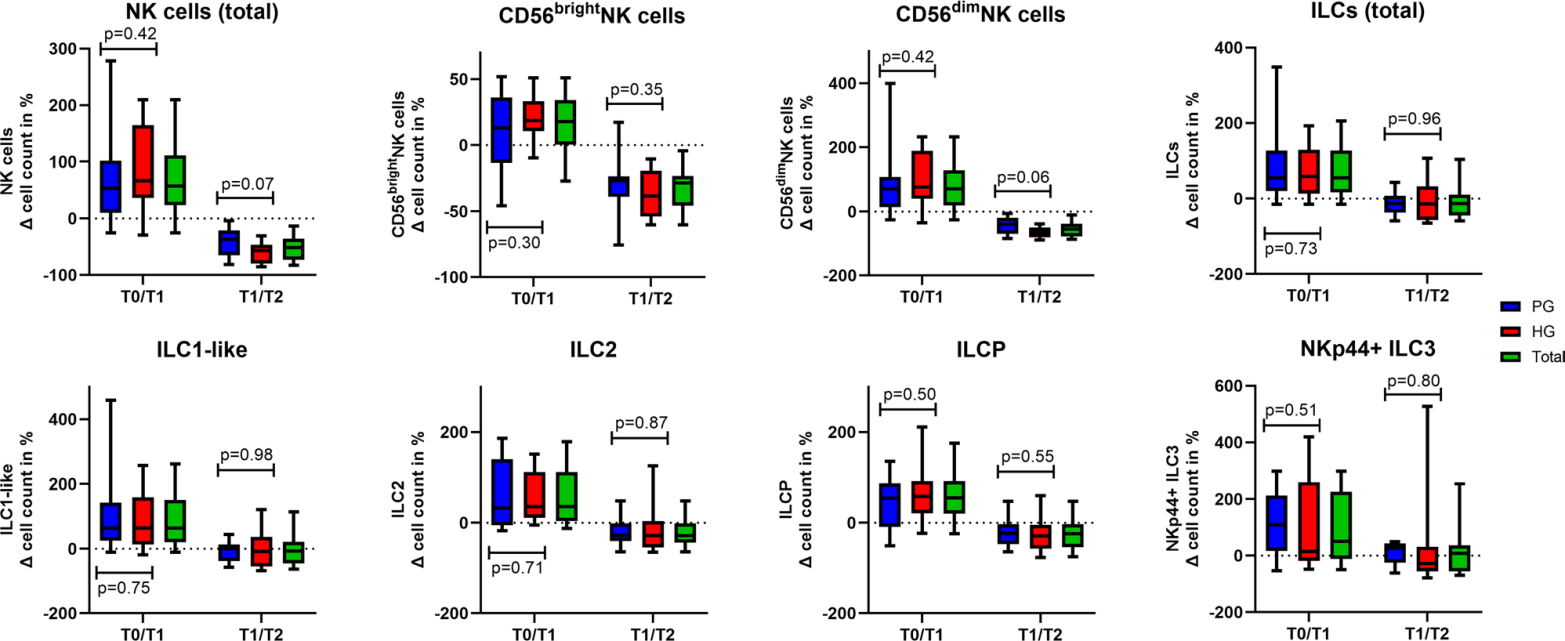
Percentual changes in cell counts from T0 to T1 and T1 to T2. The boxplots illustrate the percentual changes in cell counts of the respective immune cells. The marked line represents the median, the boxes are the interquartile ranges, and the whiskers represent the 10. to 90. percentile. P-values refer to the statistical difference of percentual immune cell count changes between HG and PG. Blue columns determine the patients changes in cell counts in percent, the red columns determine the percentual changes of healthy controls cell counts and the green columns determine the percentual changes in total for the different cell populations. P-values describes the differences between the PG and HG for the respective cells from T0 to T1 or T1 to T2. Of note, NKp44^+^ILC3 is a rare subset with low frequencies. Hence, the n varies between the different results (T0/T1 PG: *n* = 9, HG: *n* = 10; T1/T2 PG: *n* = 5, HG: *n* = 12)

A comparative analysis of the percentage changes in immune cell counts at the respective time points between the groups (Fig. 4) revealed no significant differences from T0 to T1 and T1 to T2 for total NK cells, CD56^bright^NK cells and for CD56^dim^NK cells. Additionally, there were no significant differences for total ILCs and their subpopulations, ILC1-like, ILC2, ILCP and NKp44^+^ILC3 (supplemental file S5).

### Correlation analyses

*Correlation of immune cell mobilization during exercise with age*,* gender BMI (kg/m²) and HR in the PG and HG*: All correlations are presented in Table 2. No significant correlations were found for immune cell mobilization and gender or BMI. Age correlated inversely with increased mobilization of total NK cells (*p* = 0.014), CD56^bright^NK cells (*p* = 0.003) and CD56^dim^NK cells (*p* = 0.025) in the PG. In HG it correlated with increased mobilization of CD56^bright^NK cells (*p* = 0.016). The maximal achieved HR (%) of age-predicted HR correlated moderately with the mobilization of total NK cells (PG: *p* = 0.018; HG: *p* = 0.011) and CD56^dim^NK cells (PG: *p* = 0.015; HG: *p* = 0.010) in both groups. With CD56^bright^NK cells it correlated only significantly in the HG (*p* = 0.001). For the HG the maximal reached HR (predicted to age) correlated also with the increase of ILC2 (*p* = 0.028) during the intervention.

**Table 2:** Correlation analyses for percentual changes in immune cell mobilization and clinical data

	NK cells total (∆%) T0/T1	CD56^bright^ NK cells (∆%) T0/T1	CD56^dim^ NK cells (∆%) T0/T1	ILCs total (∆%) T0/T1	ILC1-like (∆%) T0/T1	ILC2 (∆%) T0/T1	ILCPs (∆%) T0/T1	NKp44^+^ILC3 (∆%) T0/T1
**Age (PG total)**	-0.539*	-0.622**	-0.500*	-0.233	-0.209	-0.134	-0.237	0.150
**Age (HG)**	0.170	0.533*	0.101	0.174	0.174	-0.012	0.420	0.006
**Gender (PG total)**	-0.088	-0.212	-0.088	-0.018	0.000	-0.177	-0.124	-0.087
**Gender (HG)**	-0.071	-0.106	-0.124	-0.195	-0.159	-0.442	-0.244	-0.212
**BMI (kg/m** ^**2**^ **) (PG total)**	-0.250	-0.293	-0.202	-0.096	-0.029	-0.104	-0.162	0.083
**BMI (kg/m2) (HG)**	-0.176	-0.036	-0.173	-0.059	0.005	-0.015	0.370	-0.080
**Maximal reached HR (%) of maximal predicted HR (PG total)**	0.523*	0.351	0.537*	-0.018	-0.053	-0.047	-0.267	0.026
**Maximal reached HR (%) of maximal HR (predicted to age) (HG)**	0.558*	0.663**	0.559*	0.277	0.277	0.492*	-0.442	0.383

Spearman’s rank analysis (0.10–0.29 weak correlation, 0.30–0.49 moderate correlation, > 0.50 strong correlation) *p < 0.05; **p < 0.01; *n* = 20 for HG and PG

## Discussion

We investigated acute effects of a single HIIT on NK cells, ILCs, and respectively their subsets, in AYA cancer patients compared to healthy controls. In both groups, we observed significant increases of total NK cells, CD56^dim^NK cells, ILCs total, ILC1-like, ILC2 and ILCPs after exercise. These findings suggest that the previously demonstrated effects of exercise on the mobilization of NK cells in prostate cancer [13, 50], breast cancer [51] and lymphoma [12] patients also occur in adolescents and young adults with cancer. Our study also discovered a change in innate lymphoid cells due to exercise in cancer patients which has not been shown in the literature before. Correlation analyses revealed that younger age correlated with greater increase of immune cells and especially CD56^bright^NK cells in the PG. Also, a higher HR correlated with greater increase of total NK cells, CD56^dim^NK cells and CD56^bright^NK cells supporting previous studies that recognized greater effects on cell mobilization with more intense exercise in cancer patients [51] and healthy population [32]. We could not detect any changes in the expression of CD16 on the surface of CD56^dim^NK cells during exercise from T0 to T1. However, during recovery from T1 to T2, we could observe a reduced expression of CD16 on CD56^dim^NK cells for the HG. In addition, it must be pointed out, that the total cell count of circulating ILCs is very low and therefore even small changes in absolute values could contribute to a significant percentual change.

As the mean hematocrit increased considerably less than the mean value of the individual cell populations (in percent from T0 to T1), we assume that the mobilization is a real mobilization of cells into the peripheral blood and the increase of the cell counts we observed from T0 to T1 was not only due to increase of the hematocrit (supplemental file S3).

There is evidence that increased adrenaline release during exercise stimulates ß-adrenoreceptors on NK cells [18]. The fact that NK cells have the highest receptor density among immune cells could explain why NK cells are more strongly mobilized before and after an intervention than ILCs [52, 53]. However, studies suggest that immune cell mobilization might not be directly linked to ß-adrenergic stimulation in patients with prostate cancer [13] and in breast cancer survivors [41]. The stimulation of mobilization can also be explained by other factors such as increased cardiac output during exercise [41], the associated increased blood flow or the release of myokines, e.g. IL-6 [18] and IL-15 [54] by the increased muscle activity, which further activate cytotoxic immune cells. In some cases, cell counts of NK cells and some ILC subsets decreased below baseline level during recovery. Peake et al., 2017 [55] described this effect as the open window effect, in which these cells migrate to different tissues during recovery. This has been observed in mice where the bone marrow, the lung and the Peyer’s patch have been identified as migration sites for lymphocytes [53]. Whether lymphocytes also move into cancer-affected tissue is still the subject of research in humans, but has been already demonstrated for animals [18, 56]. Further research is needed to determine whether this open window effect can be observed in all types of tumor tissue after exercise. However, there is evidence that this is the case in some types of tumor tissues [57]. In addition to the decrease in absolute NK cell count during the recovery phase, a lower proportion of CD16^+^ CD56^dim^NK cells was also measured in the HG after the recovery phase. This could be due to the fact that the total number of CD56^dim^NK cells in the peripheral blood decreases, as they may migrate into the tissue, leading to the above mentioned open window effect and are therefore proportionally less detectable in the peripheral blood.

In contrast to NK cells, there is no significant decrease in ILC1-like during recovery. Cell counts of ILC1-like remain at a constant, even slightly increased level, indicating that the open window effect does not apply to this ILC subset. Our data provide evidence that exercise leads to an increased mobilization of ILCs, which has previously been observed in healthy stem cell donors [40]. Research results on the behavior of ILCs during recovery are very limited. This study has shown, that ILCPs and ILC2 cell count in peripheral blood slightly decreases during the recovery phase. Thus, like NK cells ILCPs might migrate into secondary lymphoid tissues to further differentiate into mature ILC subsets there [58]. Additionally, the behavior of NKp44^+^ILC3 needs further research. We were able to demonstrate that their pattern of changes is different between the groups. There is an increase in the number of NKp44^+^ILC3 in the PG from T0 to T1. From T1 to T2 they decrease within the PG, while there is an increase in cell count in the HG.

Although direct effects of unmodified NK cells on the tumor itself are limited due to its shielding and are still under discussion [59, 60], they have positive impact on the spread of metastases [61], immune systems [21], physical fitness, quality of life and fatigue [14]. NK cells recognize pathogens or neoplastic cells, which lead to an activation of immune systems cascades and further in their elimination [26]. Enhanced mobilization of immune cells, such as NK cells and ILC may result in an improved immune surveillance, which can further reduce the adverse effects of cancer treatment, such as increased contagiousness with viruses or fungi, and the treatment may be less burdensome. The clinical significance of increased immune cell mobilization is being discussed [62] and animal models provide insights into not only NK cell recruitment but also infiltration in solid tumors [62] and delayed tumor onset and burden [63].

Our study was an investigation of acute effects and therefore involved only a one-time intervention. However, a single intervention is unlikely to produce long-term changes. Therefore, regular exercising would be important to fully utilize the effects of immune cell mobilization and its potency. Major strengths of the study lie in the inclusion of a patient cohort that has been little studied to date, the integration of diverse innate immune cell types including ILCs that have also been little studied and the inclusion of a rigorously matched control group to classify the immune response to acute exercise in comparison to non-diseased patients. However, the special collective of patients in our study also leads to the limitation that it is difficult to generalize the results. Recruitment of our participants primarily was conducted on our AYA cancer ward, leading to a collective with different types of cancer with the majority being sarcoma patients. This sample is therefore not representing all AYA cancer types and additionally does not allow to determine results for a specific type of cancer. The generalizability of this study is therefore limited. However, the data from this pilot study provide a basis for the required in-depth studies. Due to the lack of prior maximal exercise test, intensity of the intervention was adjusted individually to the participants on the basis of subjective exhaustion, measured using the RPE scale. Consequently, even though they all indicated exercising at a high intensity based on the RPE scale, which was the primary criteria to ensure sufficient exertion, not all of them reached 85% of their age-predicted maximal HR. Possible causes for this could be previous tumor surgeries, which often lead to prolonged immobilization, limited mobility, and a related loss of muscle mass. In such cases, exertion could be more of a muscular instead of targeted endurance exertion. Additionally, maintaining wattage standardization during HIIT was challenging, with watt power sometimes being either too high for the PG or too low for HG. The altered expression of CD16 on the CD56^dim^ NK cells is only a small and indirect aspect of the functionality of immune cells. Further analyses are required in order to be able to make a definitive statement about the altered functionality of immune cells as a result of acute bout of exercise. However, the observed change in cell counts at the different time points provide a basis for further exploration of functionality changes in these cells. A further limitation of the study is that the BMI of the participants was calculated and used as a matching criterion, but the exact body composition was not recorded. This means that we cannot differentiate whether a participant with a high BMI has a particularly high proportion of muscle mass and is therefore more likely to have a high fitness level, was matched with a control whose high BMI was due to a higher proportion of fat, or vice versa. Behavioral guidelines and conditions were defined only for the intervention itself and the recovery phase, not for the pre-intervention period. Thus, we cannot eliminate possible influences from e.g., diet, caffeine, alcohol, nicotine or previous exercises.

## Conclusion

We observed that a single HIIT intervention leads to a significant increase in total NK cells, CD56^dim^NK cells, total ILCs, ILC1-like, ILC2 and ILCPs and that there is a significant decrease after recovery in total NK cells, especially CD56^dim^NK cells and some ILC subsets. These findings suggest that exercising during chemotherapy might support the immune system positively might support the immune system in AYA cancer patients, enhancing their ability to defend against pathogenic cells, recurrent neoplastic cells and may have a positive anti-tumor effect. This pilot study provides initial insights into the behavior of NK cells and ILCs in AYA patients during a single exercise session. To draw conclusions about the clinical relevance of exercise during oncological therapy, further studies are needed, which examine the behavior of immune cells during regular training and investigate the altered functionality of these immune cells.

## Electronic supplementary material

Below is the link to the electronic supplementary material.

Supplementary Material 1

## Data Availability

No datasets were generated or analysed during the current study.

## Abbreviations

AYA: Adolescents and young adults
HG: Healthy group
PG: Patient group
HIIT: High-intensity-interval training
NK cells: Natural killer cells
ILCs: Innate lymphoid cells
