# Supplementary material for: The impact of a single HIIT intervention on the mobilization of NK cells and ILCs in adolescents and young adults (AYA) undergoing cancer treatment: an interventional controlled trial
Source: BMC Cancer. 2025 Apr 14;25:689. doi: 10.1186/s12885-025-14058-3 (PMC11998356; doi:10.1186/s12885-025-14058-3)
Supplement: Supplementary file 1 — Supplementary Material 1 [file 12885_2025_14058_MOESM1_ESM.docx]

**Supplementary Material to: The impact of a single HIIT intervention on the mobilization of NK cells and ILCs in adolescents and young adults (AYA) undergoing cancer treatment: an interventional controlled trial**

**Supplemental file 1 (S1): Intervention and target values during different phases**


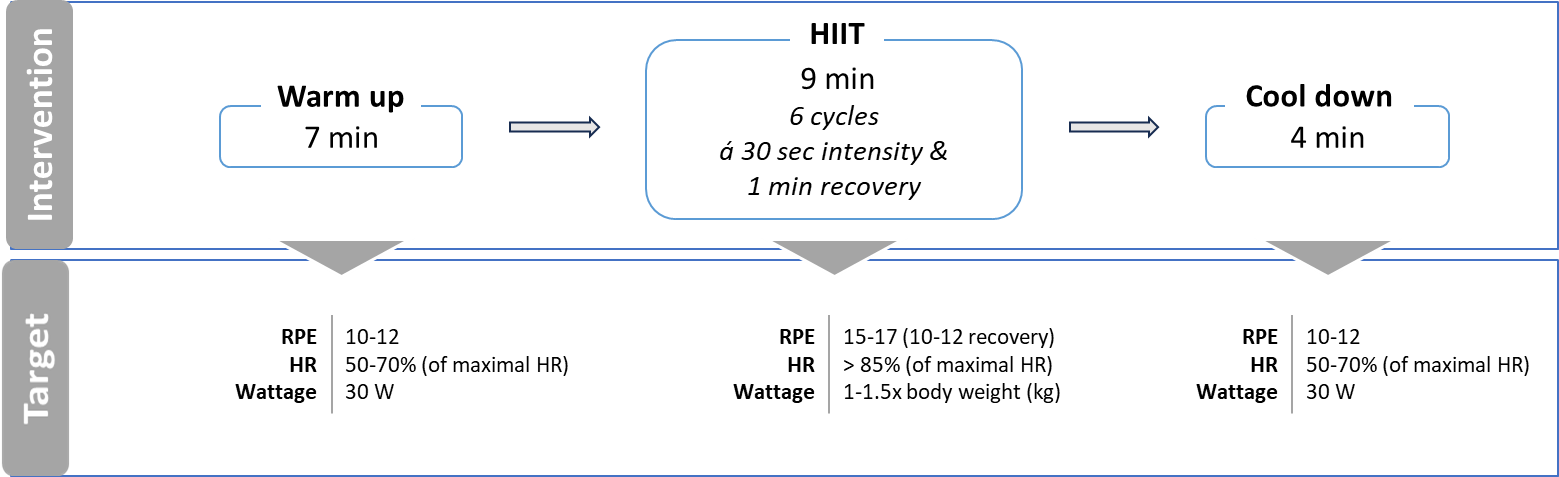


**Figure 1:** Intervention and target values during the different phases

Abbreviations: RPE= rate of perceived exertion; HR= heart rate; HIIT= high intensity interval training

**Supplemental file 2 (S2): Flow cytometry analysis**

As it was described by Bennstein et al, 2019 the isolated cells were stained by using the following FITC conjugated antibodies for the lineage panel: anti-CD1a (HI149), anti-TCRαβ (IP26), anti-TCR𝛾𝛿 (B1), anti-CD123 (6H6), anti-FceR1a (AER-37(CRA-1)), anti-CD235a (HI264) from BioLegend (CA, USA), and anti-CD3 (UCHT1), anti-CD14 (RMO52), anti-CD19 (J3-119) from Beckman Coulter (CA, USA). Also the following antibodies were further used for analysis: anti-CD94-PE/Cy7 (DX22), anti-CD56 (NCAM)-BV650^TM^ (HCD56), anti-CD117-BV421^TM^ (104D2), anti-CD294 (CRTH2)-PE/Dazzle 594^TM^ (BM16), anti-CD16-BV605^TM^ (3G8), anti-CD45-APC/Cy7 (HI30) all from BioLegend. Anti-CD336 (NKp44)-BV510^TM^(P44-8) from BD (New Jersey, USA), anti-CD127-PC5-PE/Cy5 (R34.34) and anti-CD159a (NKG2A)-APC (Z199) from Beckman Coulter.

**Supplemental file 3 (S3): Median percentual changes of NK cells, ILCs, their respective subpopulations and hematocrit from T0 to T1 and from T1 to T2**

|  | **Median T0/T1 %** | **Median T1/T2 %** |
| --- | --- | --- |
| **NK cells total** | 57.59 | -51.30 |
| **CD56^bright^NK cells** | 17.76 | -28.32 |
| **CD56^dim^NK cells** | 70.72 | -55.61 |
| **ILCs total** | 54.89 | -13.20 |
| **ILC1-like** | 63.20 | -7.40 |
| **ILC2** | 35.43 | -28.00 |
| **ILCPs** | 55.35 | -24.10 |
| **Hematocrit** | 0.01 | -0.02 |

Median percentual changes of the hematocrit, NK cells, ILCs and their respective subpopulations from T0 to T1 and from T1 to T2 for all participants (n=40)

**Supplemental file 4 (S4): Median absolute cell counts and proportion in lymphocytes of NK cells, ILCs, and their respective subpopulations for the patients and the healthy control group**

|  | **PG** | | **HG** | |
| --- | --- | --- | --- | --- |
|  | **abs. cell count**  **/µl** | **% cell count in lymphocytes** | **abs. cell count**  **/µl** | **% cell count in lymphocytes** |
| **NK cells total** |  |  |  |  |
| **T0** | 58.39 | 5.31 | 104.26 | 6.72 |
| **T1** | 98.76 | 6.76 | 185.84 | 10.00 |
| **T2** | 48.04 | 3.07 | 61.08 | 5.22 |
| **CD56^bright^NK cells** |  |  |  |  |
| **T0** | 9.16 | 0.53 | 9.94 | 1.12 |
| **T1** | 15.20 | 0.52 | 12.22 | 1.54 |
| **T2** | 5.83 | 0.43 | 8.62 | 1.06 |
| **CD56^dim^NK cells** |  |  |  |  |
| **T0** | 39.37 | 4.32 | 80.19 | 4.63 |
| **T1** | 72.98 | 5.59 | 148.99 | 6.48 |
| **T2** | 32.30 | 2.14 | 41.33 | 3.58 |
| **ILCs total** |  |  |  |  |
| **T0** | 12.28 | 1.52 | 35.40 | 1.72 |
| **T1** | 17.82 | 2.08 | 59.67 | 2.17 |
| **T2** | 13.75 | 2.28 | 50.65 | 2.13 |
| **ILC1-like** |  |  |  |  |
| **T0** | 8.51 | 1.14 | 27.25 | 1.23 |
| **T1** | 13.82 | 1.61 | 45.13 | 1.49 |
| **T2** | 10.56 | 1.88 | 41.54 | 1.65 |
| **ILC2** |  |  |  |  |
| **T0** | 2.58 | 0.23 | 5.31 | 0.30 |
| **T1** | 2.82 | 0.25 | 7.26 | 0.32 |
| **T2** | 2.08 | 0.25 | 5.19 | 0.26 |
| **ILCPs** |  |  |  |  |
| **T0** | 0.64 | 0.11 | 2.09 | 0.09 |
| **T1** | 0.82 | 0.14 | 4.23 | 0.09 |
| **T2** | 0.45 | 0.11 | 2.23 | 0.05 |
| **NKp44+ILC3** |  |  |  |  |
| **T0** | 0.001 | - | 0.007 | - |
| **T1** | 0.003 | - | 0.006 | - |
| **T2** | 0.000 | - | 0.010 | - |

Median absolute cell counts of NK cells, ILCs, and their respective subpopulations and the different measurement time points for both groups. PG total: T0 & T1 n=20; T2 n=19. HG: T0, T1, T2 n=20. Except for NKp44+ILC3: PG total: T0, T1 n=10; T2 n=11. HG: T0, T1 n=16, T2 n=13. Because the absolute cell count of NKp44+ILC3 was so small in proportion to absolute lymphocyte cell count we didn’t give the proportion for this cell type.

**Supplemental file 5 (S5): Percentage changes in cell counts of NK cells, ILCs and their subpopulations for the patient and the healthy control group.**

|  | T0/T1 | | T1/T2 | | T0/T2 | |
| --- | --- | --- | --- | --- | --- | --- |
|  | **Median** | **Range** | **Median** | **Range** | **Median** | **Range** |
| NK cells (∆%) |  |  |  |  |  |  |
| Total | 57.59 | -40.10 – 370.21 | -51.30 | -86.40 – 28.45 | -30.52 | -76.51 – 43.39 |
| PG | 53.00 | -31.11 - -82.75 | -36.60 | -82.75 – 28.45 | -15.90 | -70.84 – 42.36 |
| HG | 66.78 | -40.10 – 320.19 | -56.69 | -86.40 - -5.28 | -33.91 | -76.51 – 43.39 |
|  | **p=0.42** | | **p=0.07** | |  |  |
| CD56^bright^ NK cells (∆%) |  |  |  |  |  |  |
| Total | 17.76 | -52.50 – 69.15 | -28.32 | -87.31 – 17.96 | -17.64 | -84.11 – 44.37 |
| PG | 12.81 | -52.50 – 69.15 | -26.87 | -87.31 – 17.96 | -17.66 | -84.11 – 44.37 |
| HG | 18.63 | -28.40 – 56.76 | -38.46 | -60.44 - -4.39 | -17.02 | -41.48 – 12.42 |
|  | **p=0.30** | | **p=0.35** | |  |  |
| CD56^dim^ NK cells (∆%) |  |  |  |  |  |  |
| Total | 70.72 | -42.98 – 694.80 | -55.61 | -92.92 – 37.29 | -30.40 | -79.04 – 44.51 |
| PG | 70.72 | -36.74 – 694.80 | -39.70 | -91.00 – 37.29 | -17.23 | -69.28 – 35.26 |
| HG | 76.69 | -42.98 – 346.41 | -64.67 | -92.92 - -4.59 | -39.82 | -79.04 – 44.51 |
|  | **p=0.42** | | **p=0.06** | |  |  |
| ILCs (∆%) |  |  |  |  |  |  |
| Total | 54.89 | -25.53 – 375.64 | -13.20 | -73.66 – 644.30 | 40.14 | -42.12 – 1133.20 |
| PG | 54.89 | -25.53 – 375.64 | -13.20 | -59.50 – 163.21 | 40.14 | -32.66 – 349.31 |
| HG | 59.46 | -22.36 – 296.35 | -14.09 | -73.66 – 644.30 | 35.78 | -42.12 – 1133.20 |
|  | **p=0.73** | | **p=0.96** | |  |  |
| ILC1-like (∆%) |  |  |  |  |  |  |
| Total | 63.20 | -25.48 – 572.44 | -7.40 | -85.09 – 671.33 | 38.95 | -48.22 – 1214.53 |
| PG | 64.17 | -25.48 – 572.44 | -0.69 | -63.23 – 146.68 | 38.95 | -34.58 – 482.19 |
| HG | 63.20 | -24.06 – 320.61 | -8.92 | -85.09 – 671.33 | 42.42 | -48.22 – 1214.53 |
|  | **p=0.75** | | **p=0.98** | |  |  |
| ILC2 (∆%) |  |  |  |  |  |  |
| Total | 35.43 | -49.20 – 205.40 | -28.00 | -74.62 – 617.49 | 4.08 | -46.36 – 1268.50 |
| PG | 32.21 | -49.20 – 205.40 | -27.46 | -74.62 – 288.90 | 7.38 | -45.23 – 237.46 |
| HG | 35.93 | -16.50 – 199.50 | -28.01 | -72.37 – 617.49 | 1.02 | -46.36 – 1268.50 |
|  | **p=0.71** | | **p=0.87** | |  |  |
| ILCP (∆%) |  |  |  |  |  |  |
| Total | 55.35 | -66.85 – 402.54 | -24.10 | -81.61 – 236.54 | 3.19 | -78.12 – 254.48 |
| PG | 54.51 | -66.85 – 402.54 | -23.64 | -81.61 – 236.54 | 4.25 | -78.12 – 254.48 |
| HG | 57.94 | -25.77 – 236.22 | -29.09 | -80.10 – 214.46 | -2.57 | -44.55 – 169.37 |
|  | **p=0.50** | | **p=0.55** | |  |  |
| NKp44+ LC3 (∆%) |  |  |  |  |  |  |
| Total | 50.58 | -54.00 – 435.09 | -25.87 | -66.85 – 402.54 | -12.03 | -100.00 – 722.43 |
| PG | 109.33 | -54.00 – 298.63 | 13.00 | -100.00 – 49.14 | -21.83 | -100.00 – 164.35 |
| HG | 14.76 | -48.77 – 435.09 | -27.95 | -84.97 – 692.23 | 28.74 | -47.25 – 722.43 |
|  | **p=0.51** | | **p=0.80** | |  |  |

P-value describes the difference between the two groups from T0 to T1 and from T1 to T2 for the respective immune cells. Of note, NKp44^+^ILC3 is a rare subset with low frequencies. Hence, the n varies between the different results (T0/T1 PG: n=10, HG: n=16; T1/T2 PG: n=11, HG: n=13)
